# Uncorrected low hyperopia in young subjects induces postural instability even in those with clear visual acuity

**DOI:** 10.1371/journal.pone.0224031

**Published:** 2019-10-17

**Authors:** Byeong-Yeon Moon, Hyun Gug Cho, Dong-Sik Yu, Sang-Yeob Kim

**Affiliations:** Department of Optometry, College of Health Science, Kangwon National University, Samcheok-si, Republic of Korea; University Medical Center Goettingen, GERMANY

## Abstract

Reports have indicated the effect of myopic blur on postural stability. The objective of this study was to investigate the minimum refractive error to significantly affect postural stability through a various levels of hyperopia and myopia induced by ophthalmic lenses. Forty subjects with a mean age of 22.95 ± 2.21 years were enrolled. In all subjects, the subjective refraction with MPMVA (Maximum to Plus Maximum Visual Acuity) was performed to correct refractive error. To induce hyperopia and myopia, spherical lenses of ±1.0, ±2.0, ±3.0, ±4.0, ±5.0 and ±6.0 D were used on top of the trial frame with corrected condition as MPMVA (eyes-open with MPMVA). Under each induced-refractive error condition, general stability (ST) and sway power (SP) in frequencies by each subsystem were measured with Tetrax posturography with firm plates at patient’s upright position, after performed the measurements under the conditions of eyes-open with MPMVA and eyes-closed. ST at eyes-closed was significantly greater than that at eyes-open with MPMVA (*p* < 0.001). ST was increased significantly for induced hyperopia of -1.0 D (*p* < 0.001) with decimal visual acuity of 1.07 ± 0.17 and for induced myopia of +3.0 D (*p* = 0.011) with decimal visual acuity of 0.16 ± 0.09, as compared to that at eyes-open with MPMVA. No significant difference was observed between induced hyperopia of -6.0 D and those at eyes-closed only. SP was increased significantly at low medium-frequencies of the peripheral vestibular signals in induced hyperopia, moreover, hyperopia induced at -6.0 D lenses was significantly different compared to that at eyes-open with MPMVA. Uncorrected low hyperopia in young subjects may lead to postural instability, although they can obtain clear vision. The corrected state of ametropia, especially hyperopia, is a more important factor of appropriate visual input in stable postural adjustment than visual acuity.

## Introduction

Body equilibrium is maintained by a perceptual-motor process that integrates information from the somatosensory, vestibular, and visual systems [1]. The somatosensory system includes various receptors which generate somatic information (pressure distribution, muscle tension, muscle length and joint angle changes) for postural control [2]. The vestibular system monitors rotational head movements and provides information of the body’s position with respect to movement and gravity [2]. Although these subsystems are involved in maintaining stability of the body in space, vision plays an important role in stabilizing postural control by continuously providing information to the nervous system about the body position in the environment, especially upright position [3,4]. Several previous studies have demonstrated more significant increases in postural sway under eyes-closed conditions than under eyes-open conditions, which confirms the importance of the visual system for postural control [5–7].

Adequate postural control is essential for maintaining health and stability of the body and preventing injuries. Balance disorders are considered a growing public health problem due to their association with falls [8]. Falling increases the risk of death and disability, and may cause loss of independence, particularly in regions of the world with high proportions of the elderly population [9]. Cataract and refractive error are the most common causes of visual impairment in older adults [10], and induced poor vision reduces postural stability and significantly increases the risk of falls. Recent reports indicated that visual disability affects the QOL (quality of life) of the individual by limiting social interactions and independence [11]. With regard to the prevalence of refractive errors, Hashemi [12] performed a systematic review of refractive errors using international databases of world-wide populations according to WHO regions. Meta-regression analysis revealed an increase of myopia prevalence from 1993 (10.4%) to 2016 (34.2%) and highest prevalence of myopia and hyperopia in adults in South-East Asia and the Americas, respectively. Edwards conducted a study on the effects of refractive error on postural stability, and reported an increase in median body instability by about 51% in 50 young subjects with the added of a +5.0 D spherical lens [13]. Paulus et al. [14] reported an increase in postural instability of about 25% increase due to myopic blur induced by +4.0 D and +6.0 D lenses and a similar finding in subjects with myopic refractive error between—3.0 and—5.0 D without wearing of corrective spectacles. Recently, Diane et al. investigated the effects of optical distortion (Plano, pincushion of +10% and barrel of -10% distortion) on postural stability in myopes and emmetropes, and reported that peripheral dynamic visual stimuli was a more important factor influencing the posture of myopes versus that of emmetropes in both plano and barrel of -10% distortion [15]. Many previous studies demonstrated that refractive error significantly interfered with postural stability [13,14,16,17], but those studies had the limitation of inability to calculate the minimum refractive power that degrades postural stability; Moreover, although hyperopia is a common type of refractive error, most of those studies focused only on myopic blur [13,14,16,17]. In our earlier study, we compared the changes of postural stability under conditions of induced hyperopia and myopia [18], and showed that postural instability is also increased under hyperopia induction, which is of relevance in clinical setting; however, that study had a limitation of undetermined cause of the increased postural instability with regard to induced refractive errors. In this study, we aimed to compare the minimum refractive powers that affect postural stability for each type of refractive error by inducing various levels of hyperopic and myopic power through (±) spherical lenses. In addition, we determined the cause of increased postural instability according to the types of refractive errors through changes in postural sway power derived by Fourier transformation, when these refractive errors interfered with the subsystems involved in postural control.

## Subjects and methods

Forty subjects (25 male individuals, 15 female individuals) with mean age of 22.95 ± 2.21 years participated in the study. All participants were healthy without otoneurological, neurological, ocular diseases and related medication intake. Before postural assessment, in each participant, general eye exams including refraction, phoria test, stereopsis, and accommodative amplitude were conducted at the university’s optometry clinic. Subjects who manifested signs or symptoms associated with non-strabismic binocular dysfunction or accommodative anomaly were excluded in the main experiments based on the expected findings of normal binocular function [19]. All participants in the present study had binocular decimal acuity better than 0.9 with optical full-correction, and normal accommodative amplitude (mean of 12.01± 1.19 D by Push-up method) and stereopsis (mean of 42.00 ± 4.64 seconds of arc by Titmus fly test). We explained the purpose of this study to all participants and preformed general exams based on an oral explanation. And postural assessment were conducted in participants with written signed informed consent. The study was approved by Kangwon National University Institutional Review Board (approval number: KWNUIRB-2018-04-004-002), and conducted in accordance with the tenets of the Declaration of Helsinki.

**Fig 1:**
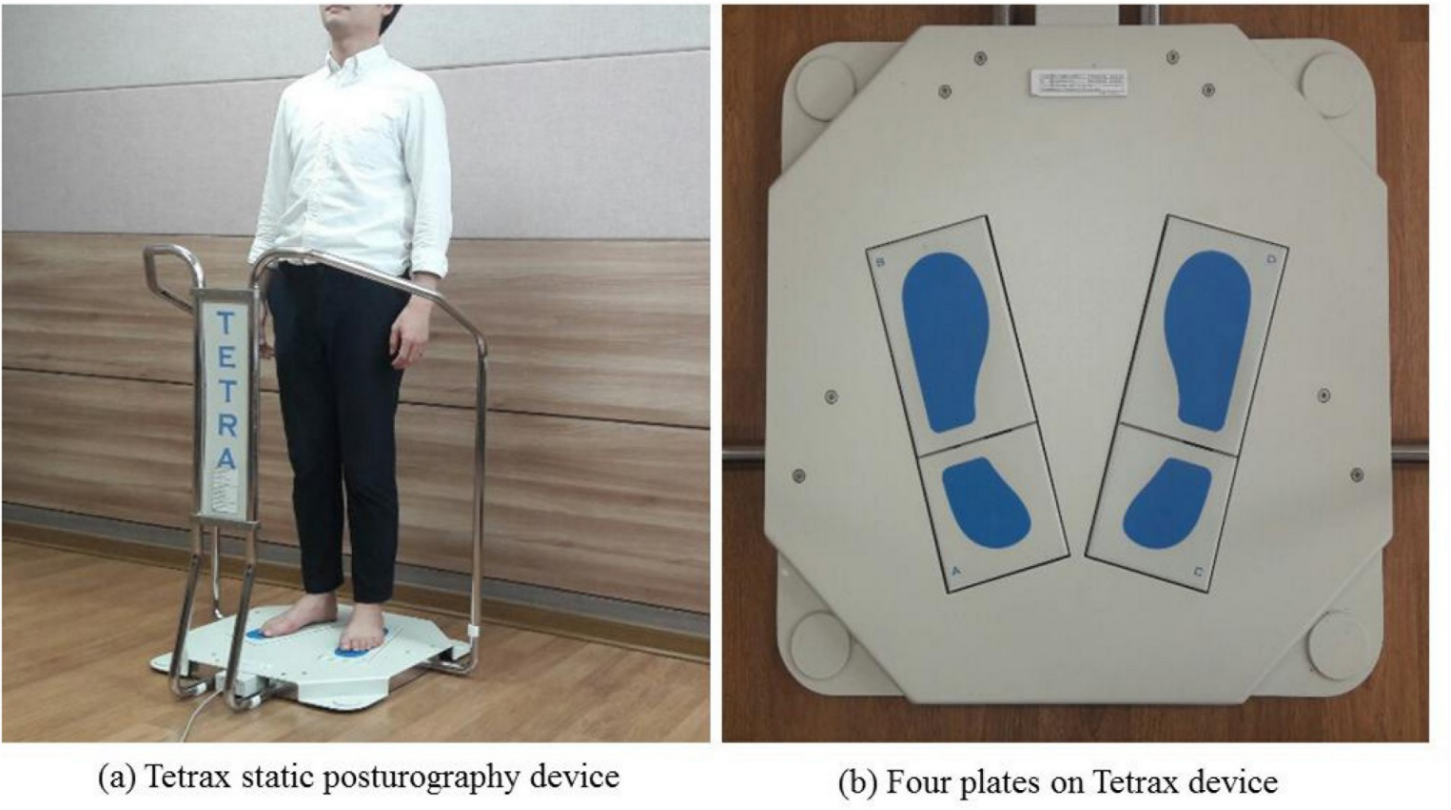
Equipment for postural assessment used in this study.

TETRAX biofeedback system (Tetrax Portable Multiple System, Tetrax Ltd., Ranmat Gan, Israel) was used to assess postural control depending on each refractive error. This equipment consists of four independent force plates that are positioned in order to measure any equilibrium disturbance from the two forefeet and two heels; the measurement points were labeled A (left foot heel), B (left fore foot), C (right foot heel), and D (right fore foot). The average value of all four measurements was obtained to evaluate the body sway in terms of displacement of the patient’s center of pressure [Fig 1]. The system has capability to analyze the general stability (ST) index of the body [20], where ST refers to the mean of oscillations recorded by four plates, with higher indices indicating postural instability. The Tetrax system also has capability to evaluate the sway power (SP) index, which is derived from Fourier spectral patterns. Fourier spectral analysis is a mathematical treatment of wave signals of body oscillations in the horizontal plane produced by the patient in order to maintain upright position [21]. SP indicates the intensity of postural sway at different frequency bands. An increase in the SP index implies dysfunction of each corresponding sensory system for postural control. The Tetrax system used in the present study subdivides the Fourier spectrum of postural sway power as follows [22]: Low frequencies (range, 0.01 to 0.1 Hz), abnormal high index may be related to visual dysfunction. Low-medium frequencies (range, 0.1 to 0.5 Hz), abnormal high index may be related to peripheral vestibular difficulties. High-medium frequencies (range, 0.5 to 0.75 Hz), abnormal high index may be related to somatosensory dysfunction. High frequencies (range, 1.0 to 3.0 Hz), abnormal high index may be related to a sign of central nervous involvement (brain damage) Clinical studies [23,24] have shown that patients with peripheral vestibular pathology typically sway at low frequency range, and the sway is intensified at medium-high range which may appear after injuries to the lower extremities, as a result of mobilization of the somato-sensory reactions mediated by the motor apparatus of the lower extremities and lower back; whereas, patients with cerebral or cerebellar lesion showed high frequency oscillations. Thus, Fourier spectral analysis of postural sway is considered as a valuable tool in clinical diagnosis, and Tetrax posturography shows high test-retest reliability [21].

To optically correct refractive errors of the subjects, subjective refraction with fogging technique was performed by the examiner using a phoropter (Ultramatic RX Master; Reichert, NY, USA) and 6-m decimal visual chart (LUCIDLC; Everview, Seoul, Korea), after non-cycloplegia objective refraction with retinoscopy (Retinocope, Welch-Allyn, MI, USA). The endpoint of refraction was determined using MPMVA (Maximum to Plus Maximum Visual Acuity) procedure [19]. Subsequently, the participants were instructed by the examiner to wear a trial frame (Trial frame TF-3, Topcon, Tokyo, Japan) with full correction and stand upright on the Tetrax device’s force plate without shoes with the arms hanging at their sides. Posture evaluations were first performed under condition of eyes-open with MPMVA and eyes-closed. Next, spherical lenses of ± 1.0, 2.0, 3.0, 4.0, 5.0 and 6.0 D were used to induce each refractive error (+ D, for induced myopia;–D, for induced hyperopia). In all induced-refractive error conditions, the subjects’ visual acuity (VA) under binocular condition was recorded. Measurements for ST and SP were randomly performed for each induced-refractive error type (myopia and hyperopia, in order) for 32 seconds according to the instructions provided in the manual. The ST index measured at each induced-refractive error type were compared at both eyes-open with MPMVA and eyes-closed conditions. Each subject was instructed to maintain their gaze on a fixed target at 6-m distance during the measurement period. The subjects rested for 1 minute during replacement of the (±) spherical lenses, and 10 minutes during presentation change of the refractive error type.

For data analysis, a paired t-test was used to compare the mean value of ST between the conditions of eyes-open with MPMVA and eyes-closed, and repeated-measures analysis of variance (ANOVA) was performed using IBM SPSS Statistics 23 to analyze the changes in ST and SP according to each refractive error. A p value < 0.05 was considered significant.

## Results

**Fig 2:**
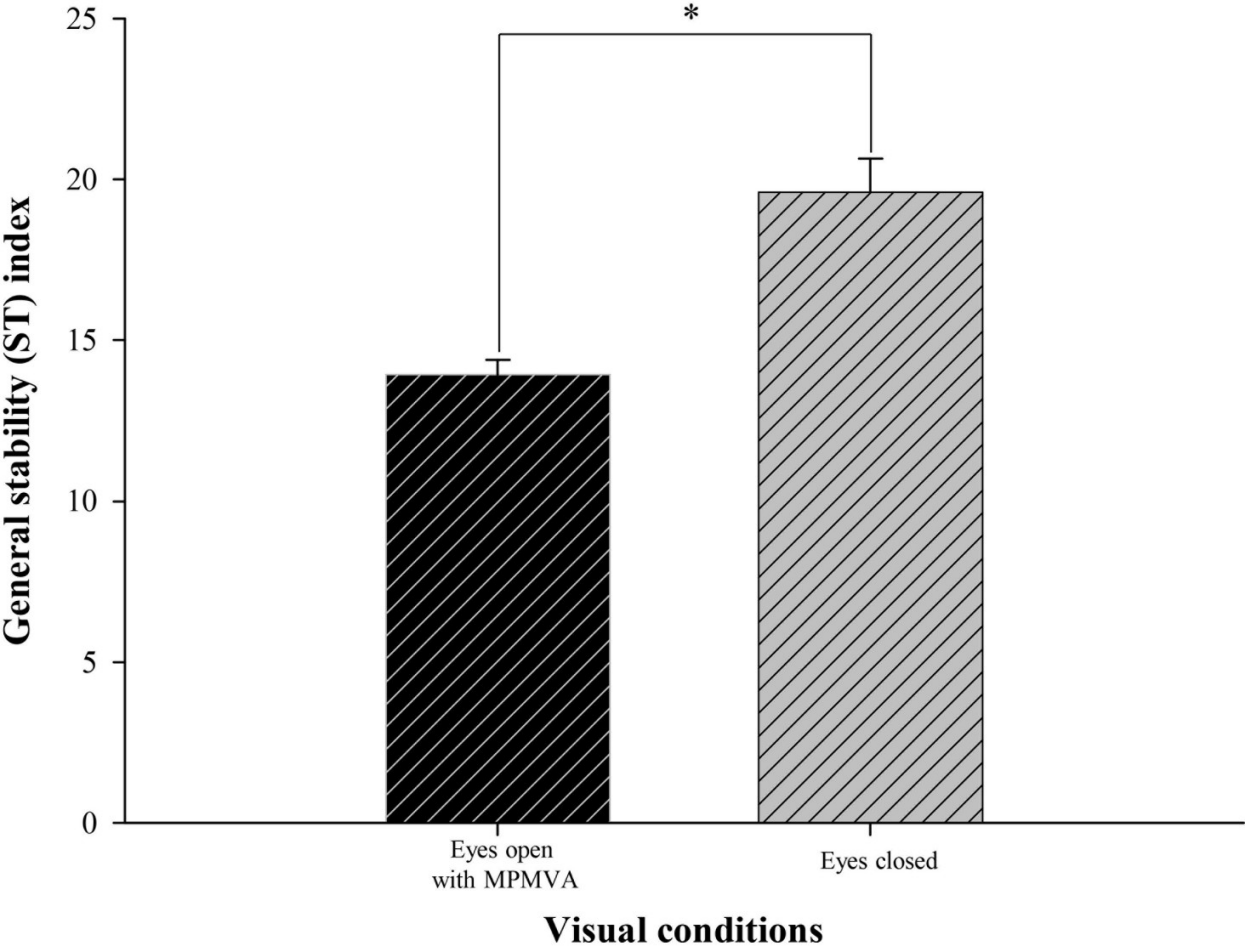
Comparison of general stability (ST) index between the eyes-open with MPMVA and eyes-closed condition. *p < 0.05; significant differences by paired t-test. Error bars indicate the standard error (SE) of the mean. MPMVA: Maximum Plus to Maximum Visual Acuity.

**Fig 3:**
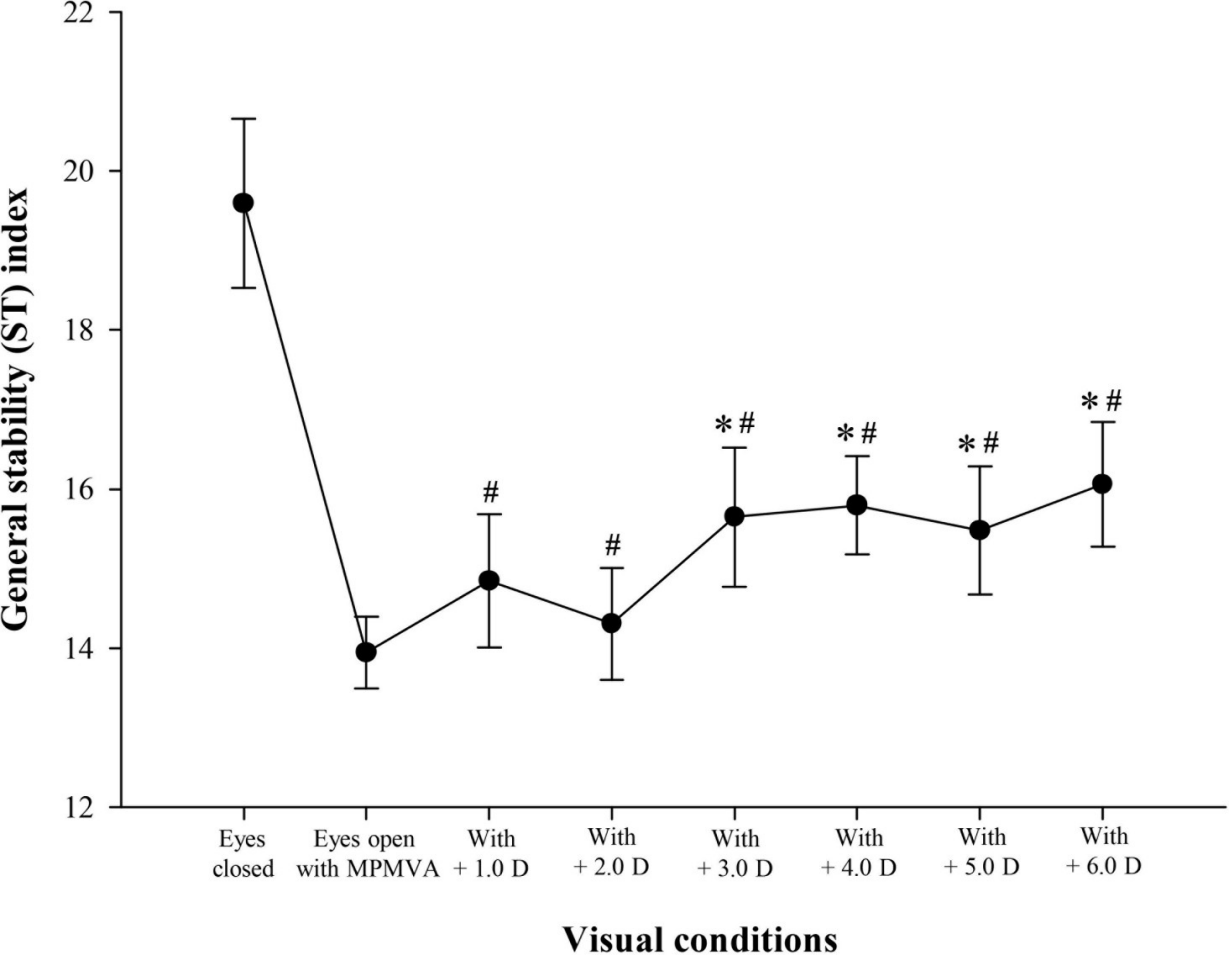
The changes of general stability (ST) index in myopia induced by (+) spherical lenses compared to those in the eyes-open with MPMVA and eyes-closed conditions. *p < 0.05; significantly different from eyes-open with MPMVA condition in myopia induced by (+) spherical lenses according to repeated measures ANOVA. ^#^*p* < 0.001; significantly different from eyes-closed condition in myopia induced by (+) spherical lenses according to repeated measures ANOVA.Error bars indicate the standard error (SE) of the mean. MPMVA: Maximum Plus to Maximum Visual Acuity.

**Fig 4:**
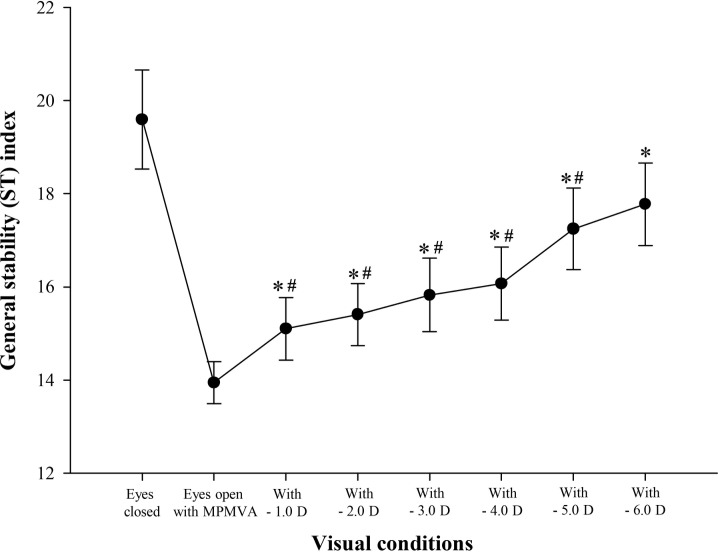
The changes of general stability (ST) index in hyperopia induced by (-) spherical lenses compared to those in the eyes-open with MPMVA and eyes closed conditions. *significantly different from eyes-open with MPMVA condition in hyperopia induced by (-) spherical lenses according to repeated measures ANOVA. ^#^significantly different from eyes-closed condition in hyperopia induced by (-) spherical lenses according to repeated measures ANOVA. Error bars indicate the standard error (SE) of the mean. MPMVA: Maximum Plus to Maximum Visual Acuity.

Difference in ST index between the eyes-open with MPMVA and eyes-closed conditions is shown in Fig 2; significantly greater value was obtained under the latter than former condition (F = -4.90; *p* < 0.001). The changes of ST according to the increment of induced-myopic and hyperopic powers between the eyes-open with MPMVA and eyes-closed condition are shown in Figs 3 and 4. In the results, significant differences of ST index compared to that at eyes-open with MPMVA was observed for induced-myopia of +3.0 D (F = 3.30 and *p* = 0.011; Fig 3) and for induced-hyperopia of -1.0 D (F = 5.98 and *p* < 0.001; Fig 4), respectively. In order to determine the level of refractive error similar to that under complete blockade of the visual input, analysis of the changes of ST index between the induced levels of refractive errors and eyes-closed condition are shown in Figs 3 and 4; no statistically significant difference between the lenses with induced hyperopia of -6.0 D and those at the eyes-closed condition only were obtained (Fig 4).

**Fig 5:**
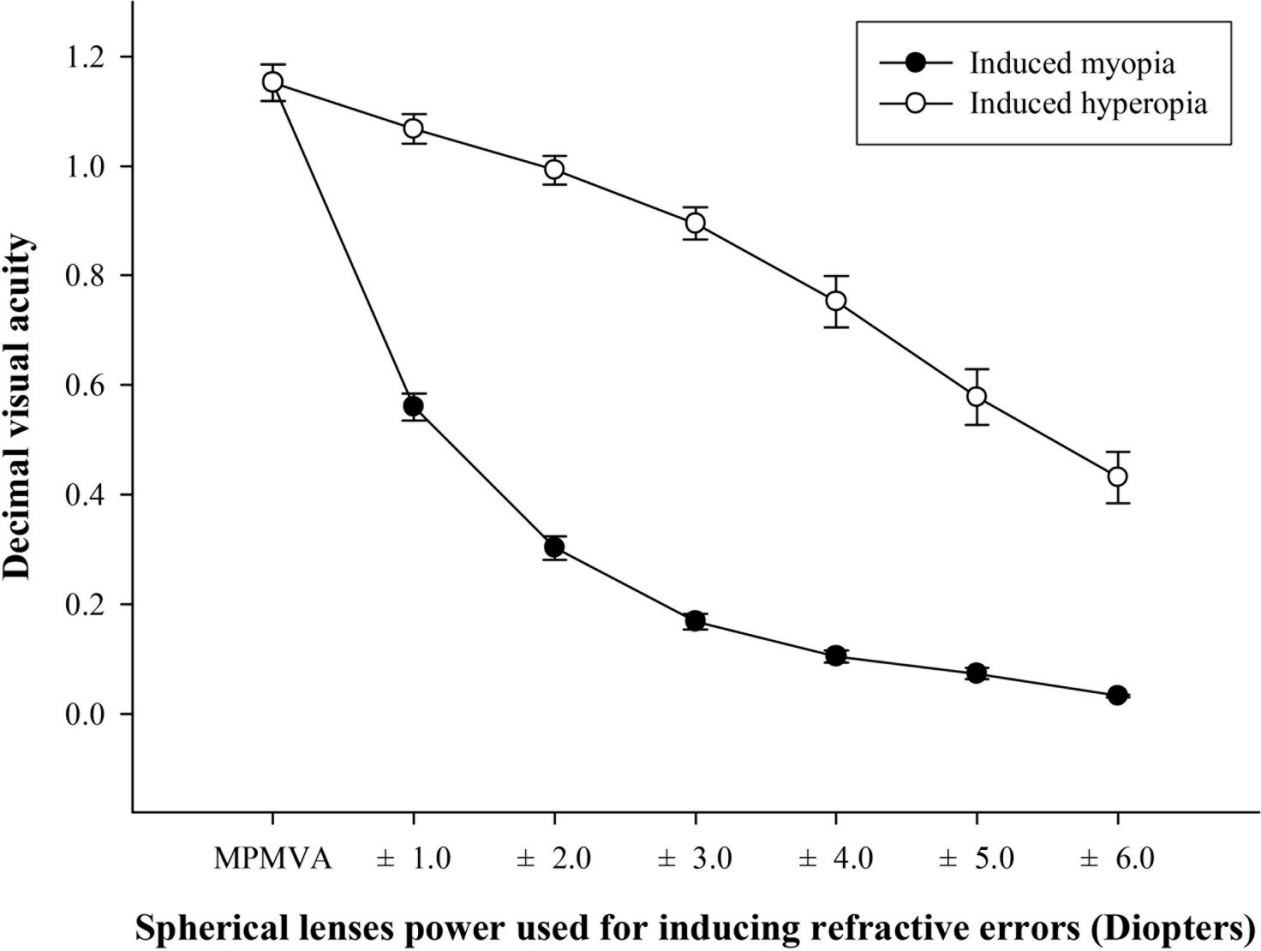
Comparison of binocular decimal visual acuity between hyperopic and myopic power induced by (±) spherical lenses. Error bars indicate standard error (SE) of the mean.

In the comparison between induced hyperopia and myopia at the same spherical power used to induce refractive errors, significant differences were obtained for the mean binocular VA at the minimum power of refractive error that affects postural stability (Fig 5).

The changes of SP index under each frequency by Fourier transformation in the induced myopia are shown in Table 1. No significant change was observed in the SP at any frequency range, despite the increase in myopic power; whereas, significant differences in the changes of SP by Fourier transformation were observed only at low-medium frequency range during hyperopia induction (Table 2).

**Table 1:** Changes of sway power (SP) index between eyes-open with MPMVA and induced-myopia at frequencies ranges derived from each subsystem by Fourier transformation.

Visual conditions	SP index in ranges of frequencies (mean ± SD) ^†^			
	Low	Low-medium	High-medium	High
Eyes open with MPMVA	22.04±10.10	8.74±3.31	2.89±1.09	0.69±1.10
With + 1.0 D	22.76±15.90	8.72±4.57	3.01±1.61	0.74±1.20
With + 2.0 D	23.50±14.69	8.43±3.93	2.92±1.32	0.76±1.37
With + 3.0 D	22.47±13.53	8.56±3.77	2.93±1.20	0.72±1.19
With + 4.0 D	22.54±12.92	8.84±3.05	3.18±1.12	0.70±1.01
With + 5.0 D	21.57±17.50	8.30±3.64	3.05±1.25	0.74±1.04
With + 6.0 D	22.09±12.74	8.87±3.52	3.24±1.44	0.77±1.03
F-value/p-value	0.09/1.0	0.36/0.90	0.68/0.67	0.56/0.76

MPMVA: Maximum Plus to Maximum Visual Acuity

^†^; Increased index in low frequency is related to visual dysfunction. Increased index in low-medium frequency is related to peripheral vestibular difficulties. Increased index in high-medium frequency is related to somatosensory dysfunction. Increased index in high frequency is related to central difficulties.

**Table 2:** Changes of the sway power (SP) between eyes-open with MPMVA and induced-hyperopia at the frequencies ranges derived from each subsystem by Fourier transformation according to increase of hyperopic power.

Visual conditions	SP index in ranges of frequencies (mean ± SD) ^†^			
	Low	Low-medium	High-medium	High
Eyes open with MPMVA	22.04±10.10	8.74±3.31^a^	2.89±1.09	0.69±1.10
With—1.0 D	21.77±12.18	8.59±3.27^a^	2.95±0.95	0.65±0.84
With—2.0 D	21.32±11.67	8.73±3.91^a^	3.03±1.12	0.71±1.10
With—3.0 D	21.61±14.80	9.72±5.13^a^	3.09±1.41	0.81±1.38
With—4.0 D	22.73±16.33	9.63±4.58^a^	3.00±1.17	0.81±1.42
With—5.0 D	22.38±11.93	9.85±4.51^a^	3.17±1.22	0.83±1.38
With—6.0 D	25.52±20.99	10.93±5.31^b^^*^	3.31±1.21	0.94±1.88
F-value/p-value	0.72/0.640	2.53/0.04	0.77/0.60	0.68/0.67

*p<0.05; significant differences by repeated-measures ANOVA

^a, b^: subgroups by LSD (Least significant difference) post-hoc analysis

MPMVA: Maximum Plus to Maximum Visual Acuity.

^†^; Increased index in low frequency is related to visual dysfunction. Increased index in low-medium frequency is related to peripheral vestibular difficulties. Increased index in high-medium frequency is related to somatosensory dysfunction. Increased index in high frequency is related to brain damages.

## Discussion

Previous studies have indicated that postural sway is increased by 20–70% when both eyes are closed, completely blocking visual input [7,14,25] which is in agreement with the findings of the current study using the Tetrax biofeedback system that postural instability was increased by about 40% at the eyes-closed condition relative to the value at the eyes- open condition (Fig 2). The collective findings of the previous and current study reconfirm that appropriate visual input is an essential factor of stable postural control. The present study primarily determined the minimum refractive power that affects postural stability, as well as variation of refractive power according to the refractive error type. We focused on the analysis of hyperopic condition rather than that of myopic condition. As shown in Figs 3 and 4, the minimum refractive power affecting ST revealed values of 1.0 D for hyperopia and 3.0 D for myopia by post-hoc analysis compared to eyes-open with MPMVA, which indicated that postural stability is more sensitively affected by uncorrected hyperopia than uncorrected myopia. This can be further supported by result that, under high hyperopia induced by -6.0 D spherical lenses, the degree of postural instability was comparable to that eyes-closed condition with complete blockade of visual inputs (Fig 4). Based on the results in Fig 5, the decimal VA differed between myopia and hyperopia for the same induced refractive powers. In subjects with induced-myopia of +3.0 D, which is the minimum myopia power that affects postural stability, the mean decimal VA was 0.17 ± 0.09, which reflects remarkable reduction. In contrast, in subjects with induced-hyperopia of -1.0 D, which is the minimum hyperopic power that affects postural stability, the mean decimal VA was of high value of 1.07 ± 0.17, which was similar to the level of VA of mean decimal value of 1.15 ± 0.21 at MPMVA condition. Myopia is a refractive error type that causes light to focus at the front of the retina, which leads to reduced VA [26], while that of hyperopia causes light rays to focus behind the retina. Fortunately, it enables focus of images to the level attained in emmetropia by automatic focusing (accommodation mechanism) [26]. This mechanism allows clear VA without optical corrections, if the amplitude of accommodation of the eye is sufficient. Previous studies described reduced VA as a major regulating factor of postural stability, which is in contrast with the findings of the present study that blurry visual input is not significant influencing factor of postural stability. Thus, uncorrected hyperopes maintain clear VA through the accommodation mechanism and may be mistaken for emmetrope; nevertheless, the visual inputs are not the same as those derived from emmetropia. Therefore, clinicians should consider the correction status of refractive errors as a criterion for appropriate visual inputs for postural stability, instead of the level of VA. To the best of our knowledge, there were no previous studies that mentioned the value of uncorrected hyperopia that affects postural stability.

The current study additionally investigated the effect of experimentally-induced increase of refractive power on the subsystems involved in postural control according to the value of refractive error. In the evaluation, we used Fourier transformation technique provided by Tetrax biofeedback system to analyze the changes of SP at four frequency ranges derived from each subsystem. The results of the present study showed that SP was significantly increased only in the low-medium frequency range of the peripheral vestibular signals; at increasing hyperopic power, there were significant differences between the experimentally-induced condition of hyperopia of -6.0 D and eyes-open with MPMVA condition (Tables 1 and 2). Clinical studies [27,28] reported excessive sway at the frequency range associated with pathological problem in the subsystem or subjects’ compensatory efforts. In particular, Taguchi [23] and Kollmitzer et al. [24] conducted studies using Tetrax system, and reported that patients with peripheral vestibular pathology typically showed sway in the range of low-medium frequencies. In our study, although we did not consider hyperopic refractive error as a serious pathological condition, high hyperopia of more than +6.0 D was a potential factor leading to abnormal postural control in patients with vestibular disorder; Moreover, our study provides important data for analyzing the postural instability of patients with low vision or amblyopia patients who might have high refractive errors.

The parasympathetic nerves of the eye originate in the Edinger-Westphal nuclei and pass through the preganglionic fibers of the oculomotor nerve to synapses in the ciliary ganglion, they control the sphincter muscle of the pupil and the ciliary muscle [26]. Here, the ciliary muscle is responsible for the control of crystalline lens. Takeda [29] noted that the imbalance of the autonomic nerve system due to the asymmetric activity of the sympathetic nerve system causes an asymmetrical blood flow of the vertebral artery, which causes the asymmetric activity of the vestibular nuclei, or inner ear, resulting in dizziness. Excessive stimulation of parasympathetic nerves leads to involuntary contraction (accommodation) of the ciliary muscle in individuals with high degree of hyperopic refractive error, and consequently, temporary imbalance of the autonomic nervous system, which may explain our result of the increase of SP at low-medium frequencies.

To summarize, the minimum refractive power that causes a reduction in postural stability was found to be low hyperopia of 1.0 D, and moderate myopia of 3.0 D, indicating that uncorrected hyperopia interferes with postural control more sensitively than blurred myopia. However, minimum refractive power for postural stability of -1.0 D, achieved maintained value of VA of 1.0 or better, which suggests that clear vision is not a decisive factor in the condition of visual inputs for stable postural control. Excessive stimulation of the parasympathetic nerves, which occurs due to a strong accommodation mechanism in individuals with high hyperopia, may interfere with the function of the vestibular system to regulate postural control. The findings of the present study apply only to healthy people in their 20s; and further study is needed to determine potential factor with greater impact in growing children with relatively incomplete physical functions and higher prevalence of latent hyperopia [30,31]. In addition, future studies to analyze postural stability in real patients with hyperopia, low vision, or amblyopia in the clinical setting are needed to validate the results of the current study using experimental model.

## Conclusion

Hyperopia with sufficient accommodative amplitude can achieve clear vision despite absence of optical correction, and contributes more significantly than myopia to reduced rate of diagnosis as refractive error and optically corrected, which results in marked reduction of VA; the relevant experts should consider this fact during postural assessment and rehabilitation of such patients. This study highlights that in young people with hyperopia with good VA, the refractive error type of the lens interferes with postural control, comparable to that of myopia. Clinicians should distinguish hyperopia from emmetropia, and perform proper optical correction for contributing stable postural control.

## Supporting information

S1 Table Sex, age, general exam. (XLSX)

S2 Table ST. (XLSX)

S3 Table Low frequency. (XLSX)

S4 Table Low-medium frequency. (XLSX)

S5 Table High-medium frequency. (XLSX)

S6 Table High frequency. (XLSX)
